# Retrospective analysis of the etiology, clinical characteristics and outcomes of community-acquired bacterial meningitis in the University Infectious Diseases Centre in Lithuania

**DOI:** 10.1186/s12879-020-05462-0

**Published:** 2020-10-07

**Authors:** E. Matulyte, S. Kiveryte, R. Paulauskiene, E. Liukpetryte, R. Vaikutyte, R. Matulionyte

**Affiliations:** 1 Department of Infectious Diseases and Dermatovenerology, Institute of Clinical Medicine, Faculty of Medicine, Vilnius University, Vilnius, Lithuania; 2 Department of Physiology, Biochemistry, Microbiology and Laboratory Medicine, Faculty of Medicine, Vilnius University, Vilnius, Lithuania

**Keywords:** Bacterial meningitis, *Neisseria meningitidis*, Invasive meningococcal disease, Vaccination, Outcome

## Abstract

**Background:**

The morbidity and mortality in community-acquired bacterial meningitis (CABM) remain substantial, and the etiology, clinical characteristics, treatment outcomes and predictors of poor prognosis must be assessed regularly. The aim of this study was to identify the distribution of etiological agents and their relationship with clinical characteristics, treatment and outcomes in this cohort of patients with CABM.

**Methods:**

Our retrospective chart review analyzed the causative microorganisms, clinical characteristics, laboratory findings, treatment and outcomes of 159 adults with CABM hospitalized in the Infectious Diseases Centre of Vilnius University Hospital from January 1, 2009 to December 31, 2016. A Glasgow Outcome Scale (GOS) score ≤ 3 was defined as unfavorable outcome. Predictors of an unfavorable outcome were identified through logistic regression analysis.

**Results:**

The median patient age was 36 (IQR 24–56), and 51.6% were male. Microbiologically confirmed causative agents were identified in 80 (50.3%) patients: *N. meningitidis* in 55 (34.6%) patients with serotype B accounting for 85% of cases, *S. pneumoniae* in 15 (9.4%), *L. monocytogenes* in 5 (3.1%) and other in 5 (3.1%). The clinical triad of fever, neck stiffness and a change in mental status was present in 59.1% of patients. Coexisting conditions and comorbidities were similar in all groups stratified by etiology. Initial antimicrobial treatment consisted of penicillin in 78 patients (49.1%) and ceftriaxone in 72 patients (45.3%). The median time in which antibiotic treatment was started was 40 min (IQR 30.0–90.0). The outcome was unfavorable in 15.7% of episodes and death occurred in 5.7% of cases and did not differ according to the causative agent. Risk factors for an unfavorable outcome were age > 65 years, coexisting pneumonia and a platelet count <150x10e9/l.

**Conclusions:**

The most common causative agent of CABM was *N. meningitidis*, with serotype B clearly dominant. Causative agents did not influence the disease outcome. The strongest risk factors for an unfavorable outcome were older age, pneumonia and a low platelet count. Since the introduction of routine vaccination against meningococcus B for infants in Lithuania in 2018, the national vaccination policy may hopefully contribute to a decrease in the incidence of serogroup B meningococcal disease in the Lithuanian population.

## Background

Community-acquired bacterial meningitis (CABM) is an impactful disease with substantial mortality and morbidity worldwide [1].

Today, CABM is usually caused by *Streptococcus pneumoniae, Neisseria meningitidis and Listeria monocytogenes* and appears with an estimated annual incidence of 0.6–4 cases per 100,000 adults. Despite substantial improvement in patient care, CABM remains a severe disease with a high risk of complications that may lead to death or severe sequelae [2–6]. Consequently, the etiology, clinical characteristics, treatment, outcomes and predictors of poor prognosis must be assessed regularly.

The highest invasive meningococcal disease notification rates in Europe were observed over the last 5 years in Lithuania (1.8 to 2.6 notifications per 100,000 population), as defined by the European Centre for Disease Prevention and Control (ECDC) surveillance data report for 2017 [7]. This high notification rate led to routine infant vaccination for meningococcus B, with the four-component capsular group B meningococcal vaccine (4CMenB) introduced into the national routine immunization program in July 2018.

The aim of this study was to identify the distribution of etiological agents and their relationship with clinical characteristics, treatment and outcomes in a cohort of university hospital patients with CABM.

## Methods

### Study center and subjects

The estimated population in Lithuania was 2.9 million people as of 2016, and Vilnius is the capital and the largest city. The Vilnius University Hospital Infectious Diseases Centre is an urban teaching hospital that provides care for approximately 2500 inpatients and 5600 outpatients annually. It is the largest of its kind in Vilnius and neighboring districts, serving 27% of the nation’s total population.

A retrospective chart review was conducted of all patients aged 18 years or older diagnosed with acute bacterial meningitis from January 1, 2009, through December 31, 2016, at the Infectious Diseases Centre of Vilnius University Hospital.

Cerebrospinal fluid (CSF) samples collected from suspected cases of bacterial meningitis were investigated by cytological examination, Gram staining and conventional microbiological culture. After cytological examination, CSF samples were centrifuged (1000 rpm) for 15 min, and sediment was used for Gram staining. One drop of well-mixed sediment was used for direct inoculation of the primary culture media – blood and chocolate agar plates. Additionally, residual pellets of sediment were inoculated into brain-heart infusion broth. Inoculated primary media and broth were incubated for 18–24 h at 35–37 °C in a 5% CO_2_ atmosphere. If no growth was observed after 24 h, the incubation time of brain-heart infusion broth was extended for an additional 48 h. Peripheral blood samples from the same patients were cultured in parallel to CSF samples. Blood samples were collected into Bact/Alert® culture media bottles and incubated in Bact/Alert 3D™ Microbial Detection System (BioMerieux, France). The isolated pathogenic bacteria were identified by the VITEK® 2 Compact automated ID/AST system (BioMerieux) and serotyped by the Pastorex™ Meningitis assay (Bio-Rad, France). Disk diffusion, the MIC gradient method and the VITEK® 2 Compact automated ID/AST system (BioMerieux) were used to test susceptibility to antimicrobials. Susceptibility results were interpreted according to the European Committee on Antimicrobial Susceptibility Testing (EUCAST) guidelines.

Bacterial meningitis of unknown origin was determined on the basis of the following inclusion criteria: a negative CSF culture with neutrophilic pleocytosis and at least one of these findings — a high (> 2.2 g/l) CSF protein level, a low (< 1.9 mmol/l) CSF glucose level, and a CSF glucose/blood glucose ratio < 0.23 — with a compatible clinical picture [8]. Bacterial meningitis was considered community-acquired if the patients had not been previously hospitalized or the onset of the disease had occurred 2 weeks after discharge from the hospital or 4 weeks after surgical treatment. Time to antibiotic therapy was calculated as time from arrival at hospital to administration of the first dose of antibiotic therapy. Two or more cases of the same serogroup in primary educational child care institutions, schools, living quarters or parallel services in a period of 4 weeks; three or more cases of the same serogroup over a period of 3 months in a determinated part of the population in a given territory or incidence ≥10/100,000 inhabitants as a result were characterized as a cluster of meningococcal disease [9, 10]. The exclusion criterion was missing medical records.

We retrospectively recorded baseline information on demographics, the time of admission, clinical characteristics, causative microorganisms, laboratory findings, treatment and outcomes.

The study was approved by the Regional Biomedical Research Ethics Committee of Vilnius University (2018-01-09, No. 158200–18–982-485).

### Statistical analysis

Categorical variables are expressed as counts (percentage), and we compared frequency distributions with Fisher’s exact test. Continuous variables are expressed as the median and inter-quartile range (IQR). Nonparametric tests (Mann-Whitney U or Kruskal-Wallis) were used to identify differences between groups in continuous outcomes. All statistical tests were two-tailed, and *P* values of less than 0.05 were considered to indicate statistical significance.

We used logistic regression to examine the association between potential predictors and the likelihood of an unfavorable outcome. Outcome was scored by the Glasgow Outcome Scale score [11]. A score of 1 on this scale indicates death; a score of 2 indicates a vegetative state (the patient is unable to interact with the environment); a score of 3 indicates severe disability (the patient is unable to live independently but can follow commands); a score of 4 indicates moderate disability (the patient is capable of living independently but unable to return to work or school); and a score of 5 indicates mild or no disability (the patient is able to return to work or school). Unfavorable outcomes were defined as death, vegetative state or severe disability (a score of 1 to 3). Univariate analysis was used to explore unadjusted associations between variables and outcome. Odds ratios and 95% confidence intervals were used to quantify the strength of these associations. A *P* value less than 0.25 and other variables of known clinical relevance (even if they are statistically insignificant) were included for further multivariable analysis. The likelihood ratio test was used to build a multivariable model. Statistical analyses were performed with use of Stata software (StataCorp, 2017; Stata Statistical Software: Release 15. College Station, TX: StataCorp LLC.).

## Results

During the study period, 167 patients were diagnosed with CABM, 159 patients were included, 8 cases excluded due to missing medical records. Among included patients, all were white, 82 (51.6%) were male, and the median age was 36 years (IQR 24–56). Microbiologically confirmed causative agent was identified in 80 patients (51.3%). The etiology was predominated by *N. meningitidis* in 55 patients (34.6%), *S. pneumoniae* in 15 patients (9.4%), and *L. monocytogenes* in 5 patients (3.1%). Among 55 *N. meningitidis* strains, serotype B was identified in 47 (85%), serotype C was identified in 1 (2%), and for the remaining 7 (13%), the serotype was not identified (Table 1). No clusters of meningococcal disease were determined during the study period.

**Table 1:** Distribution of CABM etiological agents (*n* = 159)

Causative pathogen	No. cases (%)
*Neisseria meningitidis*	55 (34.6)
- Serotype B	47 (85.5)
- Serotype C	1 (1.8)
- Not identified	7 (12.7)
*Streptococcus pneumoniae*	15 (9.4)
*Listeria monocytogenes*	5 (3.1)
Other	5 (3.1)
- *H. influenzae*	3 (1.8)
- *S. aureus*	1 (0.6)
- *K. pneumoniae*	1 (0.6)
Negative culture	79 (49.7)

*N. meningitidis* was identified in 55 patients: only in the CSF in 28 (50.9%) and in both the CSF and the blood cultures of 27 (49.1%) patients. *S. pneumoniae* was identified in 15 patients: only in the CSF culture in 4 (26.7%) patients and in both the CSF and the blood cultures in 11 (73.3%) patients. *L. monocytogenes* was identified in 5 patients: only in the CSF culture in 2 (40.0%) patients and in both the CSF and the blood cultures in 3 (60.0%) patients. Other etiological agents were *S. aureus* in one patient (in CSF and blood cultures), *S. pyogenes* in one patient (in CSF and blood cultures) and *H. influenzae* in three patients (all identified only in CSF culture). We less frequently defined the cause of bacterial meningitis in patients who had received antibiotics prior to hospital presentation than in those who had not: no organism was cultured in 59 (75%) patients with prior antibacterial treatment compared to in 20 (25%) patients who had not received antibiotics out of 79 patients with meningitis of unknown origin, *p* = 0.009.

The sex of patients with CABM was similar according to the etiological agent (female/male ratio 28/27 in the *N. meningitidis* group vs. 7/8 in the *S. pneumoniae* group vs. 4/1 in the *L. monocytogenes* group; *p* = 0.155). Patients with *N. meningitidis* meningitis were significantly younger: median age 28 years (IQR 21–50) vs. 49 years (IQR 40–64) in the *S. pneumoniae* group vs. 54 years (IQR 37–81) in the *L. monocytogenes* group; *p* = 0.155. The most common clinical symptoms at presentation were headache, reported by 134 patients (84.3%), followed by neck stiffness in 128 patients (81.0%), fever ≥38 °C in 109 patients (68.6%) and nausea in 88 patients (55.3%). The clinical triad (neck stiffness, altered mental status and fever) was present in 94 (59.1%) patients. The presence of the clinical triad was not associated with the causative agent: 38/55 (69.1%) in the meningococcal meningitis group, 6/15 (40%) in the pneumococcal meningitis group, and 3/5 (60%) in the *L. monocytogenes* meningitis group; *p* = 0.1066. The presence of hemorrhagic rash was the most common symptom in the meningococcal meningitis group, 36 (65.5%) cases, with 31 (39.2%) cases among unknown-origin meningitides and no cases in the pneumococcal, *L. monocytogenes* and other meningitis groups, *p* < 0.001. Coexisting conditions and comorbidities were similar in all groups stratified by the causative agents: 30 (55%) in 55 episodes of meningococcal meningitis, 13 (87%) in 15 episodes of pneumococcal meningitis and 3 (60%) in five episodes of listerial meningitis (*p* = 0.0625). Two of 5 *L. monocytogenes* meningitis cases occurred in adults under 50 years without specific risk factors (Table 2).

**Table 2:** Demographic and clinical characteristics of CABM cases according to the etiological agents

Characteristic	*N. meningitidis* meningitis (*n* = 55)	*S. pneumoniae* meningitis (*n* = 15)	*L. monocytogenes* meningitis (*n* = 5)	Other (*n* = 5)	Meningitis of unknown origin (*n* = 79)	Total (*n* = 159)	*P* value
Age — years median (IQR)	28 (21–50)	49 (40–64)	54 (37–81)	33 (30.5–36)	36 (23–60)	36 (24–56)	0.019
Male sex — no. (%)	27 (49.1)	8 (53.3)	1 (20.0)	5 (100.0)	41 (51.9)	82 (51.6)	0.155
Route of acquisition, n (%)							
Unknown	53 (96.4)	11 (73.3)	5 (100.0)	5 (100.0)	77 (97.5)	151 (95.0)	0.045
Otitis	1 (1.8)	3 (20.0)	0 (0.0)	0 (0.0)	2 (2.5)	6 (3.8)	
Sinusitis	1 (1.8)	1 (6.7)	0 (0.0)	0 (0.0)	0 (0)	2 (1.3)	
Headache, n (%)	50 (90.9)	10 (66.7)	4 (80.0)	4 (80.0)	66 (83.5)	134 (84.3)	0.160
Fever ≥38 °C, n (%)	39 (70.9)	9 (60.0)	3 (60.0)	4 (80.0)	54 (68.4)	109 (68.6)	0.876
Nausea, n (%)	26 (47.3)	10 (66.7)	5 (100.0)	3 (60.0)	44 (55.7)	88 (55.3)	0.181
Vertigo, n (%)	11 (20.0)	2 (13.3)	2 (40.0)	3 (60.0)	25 (31.6)	43 (27.0)	0.122
Seizures, n (%)	4 (7.3)	1 (6.7)	0 (0.0)	0 (0.0)	1 (1.3)	6 (3.8)	0.326
Hemorrhagic rash, n (%)	36 (65.5)	0 (0.0)	0 (0.0)	0 (0.0)	31 (39.2)	67 (42.1)	< 0.001
Neck stiffness, n (%)	39 (72.2)	9 (60.0)	4 (80.0)	4 (80.0)	72 (91.1)	128 (81.0)	0.007
Clinical triad, n (%)	38 (69.1)	6 (40.0)	3 (60.0)	2 (40.0)	45 (56.9)	94 (59.1)	0.1066
Comorbidities, n (%)							
• Cardiovascular disease	9 (16.4)	4 (26.7)	2 (40.0)	1 (20.0)	21 (26.6)	37 (23.3)	0.477
• Diabetes mellitus	1 (1.8)	0 (0.0)	0 (0.0)	0 (0.0)	1 (1.3)	2 (1.3) (5.0)	> 0.999
• Other^a^	7 (12.7)	3 (20.0)	0 (0.0)	0 (0.0)	12 (15.2)	22 (13.8)	0.867

^a^Other comorbidities: alcoholism (*n* = 3 (1.8%)), a history of cancer (*n* = 1 (0.6%)), rheumatoid arthritis (*n* = 1 (0.6%)), cerebrovascular disease (*n* = 11 (6.9%)), opioid addiction (*n* = 2 (1.2%)), multiple sclerosis (*n* = 1 (0.6%)), hepatitis C (*n* = 2 (1.2%)), and gastrointestinal disease (*n* = 1 (0.6%))

We observed a median CSF white cell count of 1706.34 cells per μl (IQR 160.83–5120.0), a median CSF protein of 2.76 g/l (IQR 1.23–5.44) and a median CSF glucose of 2.1 mmol/l (IQR 0.30–3.20). The highest CSF white cell count was in the other (*H. influenzae* – 3 patients, *S. aureus* – 1 patient, *S. pyogenes* – 1 patient) meningitis group, and the lowest was in the *L. monocytogenes* meningitis group. The median CSF white cell count was 3968.16 cells per μl (IQR 1021.33–5290.75) in the other meningitis group, 1706.67 cells per μl (IQR 143.30–7381.33) in the *N. meningitidis* group, 2805.0 cells per μl (IQR 396.90–5333.33) in the *S. pneumoniae* group and 225.0 cells per μl (IQR 102.50–592) in the *L. monocytogenes* group, but no significant difference was found (*p* = 0.227). The highest CSF protein level was in the other meningitis group, and the lowest CSF glucose level was in the *N. meningitidis* and *S. pneumoniae* groups. The median CSF protein level and the median CSF glucose level were 6.55 g/l (IQR 3.48–89.50) and 1.80 mmol/l (IQR 0.30-NA) in the other meningitis group, 4.10 g/l (IQR 1.47–6.54) and 0.55 mmol/l (IQR 0.30–2.79) in the *N. meningitidis* group, 3.91 g/l (IQR 2.58–6.14) and 0.55 mmol/l (IQR 0.30–3.70) in the *S. pneumoniae* group, and 2.29 g/l (IQR 0.94–5.06) and 1.22 mmol/l (IQR 0.67–2.40) in the *L. monocytogenes* group; *p* = 0.013 and *p* = 0.007, respectively. The highest serum white blood cell (WBC) count was noted in the *S. pneumoniae* group. The median WBC count was 19.52 x10e9/l (IQR 14.06–25.48) in the *S. pneumoniae* group, 18.30x10e9/l (IQR 14.20–25.70) in the *N. meningitidis* group, and 8.40x10e9/l (IQR 6.89–8.49) in the *L. monocytogenes* group; *p* < 0.001. The lowest platelet (PLT) count and the highest level of C-reactive protein (CRP) were more commonly seen in the *N. meningitidis* group. The median PLT count and the median CRP level were 172.0 x10e9/l (IQR 129–227.0) and 175.32 g/l (IQR 125.24–217.0) in the *N. meningitidis* meningitis group, 204.0 x10e9/l (IQR 161.0–267.0) and 138.49 g/l (IQR 85.0–186.07) in the *S. pneumoniae* group, and 288.0 x10e9/l (IQR 198.0–298.50) and 65.29 g/l (IQR 21.07–73.70) in the *L. monocytogenes* group; *p* = 0.048 and *p* < 0.001, respectively. The level of blood glucose was significantly lower in patients with *L. monocytogenes* meningitis. The median blood glucose was 6.50 mmol/l (IQR 5.55–7.78) in the *L. monocytogenes* group, 7.49 mmol/l (IQR 6.65–9.87) in the *N. meningitidis* group, and 8.44 mmol/l (IQR 7.37–11.23) in the *S. pneumoniae* group; *p* = 0.023 (Table 3).

**Table 3:** Laboratory findings of CABM cases according to the etiological agents

Laboratory finding	*N. meningitidis* meningitis (*n* = 55)	*S. pneumoniae* meningitis (*n* = 15)	*L. monocytogenes* meningitis (*n* = 5)	Other (*n* = 5)	Meningitis of unknown origin (*n* = 79)	Total (*n* = 159)	*P* value
CSF white cell count – cells per μl, n, median (IQR)	53, 1706.67 (143.30–7381.33)	14, 2805,0 (396.90–5333.33)	5, 225.0 (102.50–592.0)	4, 3968.16 (1021.33–5290.75)	76, 1655.66 (78.50–5013.33)	152, 1706.34 (160.83–5120.0)	0.227
CSF protein – g/l, n, median (IQR)	49, 4.10 (1.47–6.54)	13, 3.91 (2.58–6.14)	5, 2.29 (0.94–5.06)	4, 6.55 (3.48–89.50)	64, 1.95 (0.80–4.53)	135, 2.76 (1.23–5.44)	0.013
CSF glucose – mmol/l, n, median (IQR)	48, 0.55 (0.30–2.79)	12, 0.55 (0.30–3.70)	5, 1.22 (0.67–2.40)	3, 1.80 (0.30-NA)	63 2.50 (1.40–3.50)	131, 2.1 (0.30–3.20)	0.007
WBC – x10e9/l, median (IQR)	18.30 (14.20–25.70)	19.52 (14.06–25.48)	8.40 (6.89–8.49)	16.32 (14.06–23.54)	14.0 (9.10–20.99)	16.1 (9.70–23.8)	< 0.001
PLT – x10e9/l, median (IQR)	172.0 (129–227.0)	204.0 (161.0–267.0)	288.0 (198.0–298.50)	175.0 (119.0–238.0)	223.0 (153.0–277.0)	203.0 (142.0–266.0)	0.048
CRP – mg/l, n, median (IQR)	55, 175.32 (125.24–217.0)	15, 138.49 (85.0–186.07)	5, 65.29 (21.07–73.70)	5, 137.39 (31.96–220.87)	78, 127.71 (55.73–184.0)	158, 147.50 (78.43–197.21)	< 0.001
Blood glucose – mmol/l, n, median (IQR)	54, 7.49 (6.65–9.87)	14, 8.44 (7.37–11.23)	5, 6.50 (5.55–7.78)	4, 6.78 (6.33–7.41)	72, 6.80 (5.95–8.47)	149, 7.40 (6.33–8.95)	0.023

Initial antimicrobial treatment most frequently consisted of penicillin in 78 patients (49.1%) and ceftriaxone in 72 patients (45.3%). The median time in which antibiotic treatment was started was 40 min (IQR 30.0–90.0) and it did not differ significantly according to the causative agent of CABM: 30.0 min (IQR 15.0–60.0) in the meningococcal meningitis group, 50 min (IQR 30.0–100.0) in the pneumococcal meningitis group and 100.0 min (IQR 50.0–370.0) in the listerial meningitis group; *p* = 0.067. The median duration of antibiotic therapy was 14 days (IQR 11.0–16) and it was significantly longer in the *L. monocytogenes* group (median 26 days, IQR 21.0–29.0) than in the *N. meningitidis* group (13 days, IQR 10.0–16.0) and the *S. pneumoniae* group (17 days, IQR 12.0–22.0) (*p* = 0.004). Penicillin-resistant *N. meningitidis* was identified in 2 (3.6%) patients, and penicillin-resistant *S. pneumoniae* was identified in 5 (33.3%) patients. The patients with *N. meningitidis* meningitis more commonly needed hospitalization in the intensive care unit (ICU) (85.5% in the meningococcal meningitis group vs. 73.3% in the pneumococcal meningitis group vs. 60% in the *L. monocytogenes* meningitis group; *p* < 0.001). One hundred six (66.7%) patients with CABM were treated in the ICU. The duration of hospitalization in the ICU was significantly longer in the *S. pneumoniae* group (median 6.50 days, IQR 4.50–10.0) than in the *N. meningitidis* group (4 days, IQR 3–5) and the *L. monocytogenes* group (2 days, IQR 1.0-NA) (*p* = 0.017). The total duration of hospitalization was significantly longer in the *L. monocytogenes* group (median 28 days, IQR 21.0–30.5 vs. *N. meningitidis* group, 15 days, IQR 12.0–19.0 vs. *S. pneumoniae* group, 20 days, IQR 13.0–25.0; *p* = 0.007) (Table 4).

**Table 4:** Hospitalization characteristics and outcomes of CABM patients

Characteristic	*N. meningitidis* meningitis (*n* = 55)	*S. pneumoniae* meningitis (*n* = 15)	*L. monocytogenes* meningitis (*n* = 5)	Other (*n* = 5)	Meningitis of unknown origin (*n* = 79)	Total (*n* = 159)	*P* value
Time to antibiotic therapy, minutes, median (IQR)	30.0 (15.0–60.0)	50 (30.0–100.0)	100.0 (50.0–370.0)	30.0 (30.0–75.0)	90.0 (30.0–90.0)	40.0 (30.0–90.0)	0.067
Duration of antibiotic therapy, days, median (IQR)	13.0 (10.0–16.0)	17.0 (12.0–22.0)	26.0 (21.0–29.0)	15.0 (11.0–17.0)	13.0 (11.0–16.0)	14.0 (11.0–16.0)	0.004
The need of intensive care, n (%)	47 (85.5)	11 (73.3)	3 (60.0)	1 (20.0)	44 (55.7)	106 (66.7)	< 0.001
Duration of hospitalization in Intensive care unit – days, median (IQR)	40, 4 (3–5)	10, 6.50 (4.50–10.0)	3, 2 (1.0 -NA)	1, NA	51, 3 (2.0–4.0)	105, 3 (3–5)	0.017
Total duration of hospitalization – days, median (IQR)	15 (12.0–19.0)	20 (13.0–25.0)	28 (21.0–30.5)	15 (11.0–19.5)	14 (12.0–18.0)	15 (12–19)	0.007
Unfavorable outcome – n (%)	11 (20.0)	3 (20.0)	2 (40.0)	1 (20.0)	8 (10.0)	25 (15.7)	0.162
Death – n (%)	5 (9.1)	1 (6.7)	0 (0.0)	0 (0.0)	3 (3.8)	9 (5.7)	0.452

Among 159 patients with CABM, 150 (94.3%) patients survived, and 9 (5.7%) patients died. The mortality rate did not vary depending on the causative organism; it was 9.1% for meningococcal meningitis compared with 6.7% for pneumococcal meningitis and 3.8% for meningitis of unknown origin (*p* = 0.452) (Table 4). Neurological sequelae occurred in 9 (5.7%) patients, consisting of polyneuropathy in 6 (3.9%), paresis in 2 (1.2%) and plegia in 1 (0.6%) patient. The outcomes were scored by the Glasgow Outcome Scale (GOS) score: 9 (5.7%) cases were categorized as GOS 1, no cases as GOS 2, 16 (10.1%) cases as GOS 3, 36 (22.6%) cases as GOS 4 and 98 (61.6%) cases as GOS 5 at discharge. A univariate analysis was performed to explore unadjusted associations between the features at presentation and the outcome. The unfavorable outcome (GOS score of ≤3) did not differ significantly according to the causative agent. Characteristics associated with an unfavorable outcome included age > 65 years (OR, 5.71; 95% CI, 2.16–15.12; *p* < 0.001), cardiovascular disease (OR, 4.17; 95% CI, 1.50–11.61; *p* = 0.006), headache (OR, 0.31; 95% CI 0.12–0.82; *p* = 0.019), pneumonia (OR, 9.29; 95% CI 3.55–24.26; *p* < 0.001), PLT count < 150 x10e9/l (OR, 3.65; 95% CI 1.51–8.83; *p* = 0.004), and the level of CSF glucose > 2.48 mmol/l (OR, 0.25; 95% CI 0.08–0.83; *p* = 0.022) (Table 5). Time to antibiotic therapy was not associated with unfavorable outcome at discharge (Table 6). A multivariable model was further established to identify factors associated with an unfavorable outcome. Characteristics associated with a higher risk of unfavorable outcome by multivariate modeling included age > 65 years (OR, 6.50; 95% CI 1.54–27.39; *p* = 0.011), coexisting pneumonia (OR, 7.07; 95% CI 2.33–21.44; *p* = 0.001) and reduced PLT count < 150 x10e9/l at presentation (OR, 3.08; 95% CI 1.05–9.05; *p* = 0.041) (Table 7).

**Table 5:** Association of demographic, etiological and clinical factors with an unfavorable outcome in patients with CABM

	Favorable outcome	Unfavorable outcome	Univariate OR (95% CI)	p
Gender, male (%)	68/134 (50.8)	14/25 (56.0)	1.23 (0.52–2.92)	0.630
Age, 65+ (%)	14/134 (10.5)	10/25 (40.0)	5.71 (2.16–15.12)	< 0.001
Etiological agent (%)				
*N. meningitidis*	44/134 (32.8)	11/25 (44.0)	2.22 (0.83–5.94)	0.113
*S. pneumoniae*	12/134 (9.0)	3/25 (12.0)	2.22 (0.51–9.56)	0.285
*L. monocytogenes*	3 (2.2)	2 (8.0)	5.92 (0.86–40.87)	0.071
Other	4 (3.0)	1/25 (4.0)	2.22 (0.22–22.35)	0.499
Meningitis of unknown origin (%)	71 (53.0)	8/25 (32.0)	Ref.	–
Comorbidities (%)				
No	90/134 (67.2)	8/25 (32.0)	Ref.	
CVD	27/134 (20.2)	10/25 (40.0)	4.17 (1.50–11.61)	0.006
CD	2/134 (1.5)	0/25 (0.0)	1.0	–
Other	15/134 (11.2)	7/25 (28.0)	5.25 (1.66–16.62)	0.005
The need of intensive care (%)	91/134 (67.9)	15/25 (60.0)	0.71 (0.29–1.71)	0.443
Headache (%)	117/134 (87.31)	17/25 (68.0)	0.31 (0.12–0.82)	0.019
Fever (%)	96/134 (71.6)	13/25 (52.0)	0.43 (0.18–1.02)	0.056
Nausea (%)	72/134 (53.7)	16/25 (64.0)	1.53 (0.63–3.71)	0.345
Vertigo (%)	36/134 (26.9)	7/25 (28.0)	1.06 (0.41–2.75)	0.907
Seizures (%)	4/134 (3.0)	2/25 (8.0)	2.83 (0.49–16.33)	0.246
Hemorrhagic rash (%)	59/134 (44.0)	8/25 (32.0)	0.60 (0.24–1.48)	0.267
Coexisting conditions: pneumonia (%)	14/134 (10.5)	13/25 (52.0)	9.29 (3.55–24.26)	< 0.001
nasopharingitis (%)	4/134 (3.0)	1/25 (4.0)	1.35 (0.15–12.65)	0.790
Platelet count – x 10e9/l (%)				
< 150	30/134 (22.4)	13/25 (52.0)	3.65 (1.51–8.83)	0.004
150–450	101/134 (75.4)	12/25 (48.0)	Ref.	–
> 450	3/134 (2.2)	0/25 (0.0)	–	–
WBC – x 10e9/l (%)				
< 10	33/134 (24.6)	7/25 (28.0)	Ref.	
10–20	53/134 (39.6)	12/25 (48.0)	1.07 (0.38–2.99)	0.901
> 20	48/134 (35.8)	6/25 (24.0)	0.59 (0.18–1.91)	0.379
CRP – > 57 mg/l (%)	110/134 (82.1)	20/25 (83.3)	1.09 (0.34–3.48)	0.883
Blood glucose – mmol/l (%)				
< 3.9	1/126 (0.8)	1/23 (4.4)	6.58 (0.39–112.40)	0.193
3.9–7.8	79/126 (62.7)	12/23 (52.2)	Ref.	–
7.8–11.1	35/126 (27.8)	6/23 (26.1)	1.13 (0.39–3.25)	0.823
> 11.1	11/126 (8.7)	4/23 (17.4)	2.39 (0.66–8.74)	0.187
CSF White cell count – cells per μL (%)				
< 100	30/127 (23.6)	4/25 (16.0)	0.67 (0.20–2.24)	0.513
100–999	25/127 (19.7)	8/25 (32.0)	1.60 (0.58–4.39)	0.361
1000-10,000	60/127 (47.2)	12/25 (48.0)	Ref.	–
> 10,000	12/127 (9.5)	1/25 (4.0)	0.42 (0.05–3.51)	0.421
CSF neutrophils – > 50% (%)	98/114 (86.0)	20/23 (87.0)	1.09 (0.29–4.09)	0.900
CSF protein – > 0.45 g/l (%)	100/112 (88.5)	22/22 (100.0)	NA	–
CSF glucose – > 2.48 mmol/l (%)	50/109 (45.9)	4/22 (18.2)	0.26 (0.08–0.83)	0.022

**Table 6:** Association of the time to antibiotic therapy and outcomes in patients with CABM

Antibiotic therapy initiated, n (%)	Favorable outcome (*n* = 134)	Unfavorable outcome (*n* = 25)	Total (*n* = 159)	*P* value
0–1 h after admission	97 (72.4)	14 (56.0)	111 (69.8)	0.307
1–2 h after admission	20 (14.9)	6 (24.0)	26 (16.4)	
2–4 h after admission	9 (6.7)	3 (12.0)	12 (7.5)	
4–6 h after admission	2 (1.5)	1 (4.0)	3 (1.9)	
> 6 h after admission	4 (4.5)	1 (4.0)	7 (4.4)

**Table 7:** Multivariable analysis of factors associated with an unfavorable outcome in patients with CABM

	Univariable odds ratio for unfavorable outcome (95% CI)	Multivariable OR for unfavorable outcome (95% CI)	*P* value
Age, 65+ (%)	5.71 (2.16–15.12)	6.50 (1.54–27.39)	0.011
Comorbidities			
No	Ref.	Ref.	
CVD	4.17 (1.50–11.61)	1.48 (0.34–6.42)	0.598
Other	5.25 (1.66–16.62)	3.42 (0.87–13.43)	0.079
Fever	0.43 (0.18–1.02)	0.40 (0.14–1.17)	0.094
Coexisting conditions: pneumonia	9.29 (3.55–24.26)	7.07 (2.33–21.44)	0.001
Platelet count – x10e9/l			
< 150	3.65 (1.51–8.83)	3.08 (1.05–9.05)	0.041
150–450	Ref.	Ref.	–

A description of multivariable model: *N* = 154, LR chi2 = 41.19, d.f = 6, *p* < 0.0001, Hosmer-Lemeshow chi2 = 2.66, d.f. = 6, *p* = 0.850. Sensitivity = 40.00%, Specificity = 96.90%. Positive predictive value = 71.43%. Negative predictive value = 89.29%. Area under ROC curve = 0.8440

## Discussion

CABM remains an important public health problem, may lead to serious sequelae and has a high impact on patients’ quality of life [1]. The etiology, clinical characteristics, treatment outcomes and predictors of poor prognosis of CABM must be assessed regularly due to the substantial morbidity and mortality [2–5]. Therefore, the aim of our study was to retrospectively identify the distribution of etiological agents and their relationship with clinical characteristics, treatment and outcomes in a cohort of patients with CABM.

The most common causative agent of CABM in our study was *N. meningitidis* (34.6%). Similarly, *N. meningitidis* was noted as the most frequent pathogen in just a few studies: Gjini et al. found that *N. meningitidis* was responsible for meningitis in 84% of cases in young adults in England and Wales and Sigurdardottir B. et al. study showed that *N. meningitidis* was a dominant causative agent in 56% of CABM in Iceland [12, 13]. However, this finding is in contrast with other studies in which *S. pneumoniae* was the major etiological agent of CABM in adults. *S. pneumoniae* is now the most common cause of CABM in all age groups (except neonatal meningitis, in which *Streptococcus agalactiae* and *E. coli* are the most common pathogens) and is diagnosed in 50–70% of cases despite the widespread availability of vaccines. Furthermore, *N. meningitidis* in other studies is the second-most common etiological agent in CABM and is important as a causative agent of outbreaks in Sub-Saharan Africa. Finally, it should be noted that meningococcal meningitis is an urgent condition in which early accurate diagnosis and rapid administration of appropriate antibacterial treatment are the key factors for the beneficial clinical outcome [3, 14–16].

Serotype B is responsible for most cases of invasive meningococcal disease, especially in children under the age of five, and serotypes C, Y, and W are predominantly responsible for invasive meningococcal disease cases in older age groups [4, 7, 17–19]. In our cohort, we observed that serotype B was responsible for the majority of meningococcal meningitis cases. Unlike most previously reported outbreaks of invasive meningococcal disease that were associated with serotype B [20], we found that all our cases of meningococcal meningitis were sporadic.

According to other studies, *L. monocytogenes* is the third most common causative agent of CABM and is associated with older age and immunodeficiency [15, 21–24]. In our study, *L. monocytogenes* meningitis was the third most common etiological factor and 40% of *L. monocytogenes* meningitis in our study occurred in adults under the age of 50 without specific risk factors. Nevertheless, we cannot conclude about the risk factors of *L. monocytogenes* meningitis in our study due to a small number of cases.

CSF culture is the gold standard to diagnose bacterial meningitis [25]. However, a major proportion of CABM causative agents were not identified. We hypothesize that it could be associated with antibiotic therapy before admission to the hospital. Furthermore, bacterial meningitis cases caused by unidentified etiological agents could probably be *N. meningitidis,* and this finding emphasized the possible underestimation of the burden of meningococcal meningitis. Polymerase chain reaction becomes an important diagnostic tool for CABM and would be a useful tool for causative agent detection several days after the start of antibiotic treatment [26].

Considering the age of patients, the most common established etiological agents of CABM in adults are *S. pneumoniae, N. meningitidis* and *L. monocytogenes*; in children beyond the neonatal age, the most common are *N. meningitidis* and *S. pneumoniae* [15]. Unsurprisingly, younger age was significantly more common in patients with *N. meningitidis* meningitis in our cohort. We did not find associations between sex and causative factors.

The clinical presentation of CABM varies and depends on the patient’s age, coexisting conditions and comorbidities [15, 27–31]. Our study revealed the most common signs of CABM – headache, fever ≥38 °C and nausea. Additionally, 59.1% of patients with CABM had the clinical triad, and it had no associations with the etiological factor. In a 20-year retrospective study conducted in Iceland, in which 51% of patients had the clinical triad, the most common pathogen was *N. meningitidis,* similar to our study [13]. Therefore, we consider that more patients with the clinical triad were found in our study due to the dominant etiological agent, *N. meningitidis*. However, multiple other studies have been performed on the clinical characteristics of adults with bacterial meningitis, showing that the clinical triad is reported in 41–51% of patients, less frequently than in our study [14]. Accordingly, the absence of the clinical triad cannot be used to exclude the possibility of bacterial meningitis. In our study, we were able to demonstrate only one etiological association between the presence of hemorrhagic rash and *N. meningitidis.* Previous studies also concluded that rashes occur more often in patients with *N. meningitidis* meningitis [31, 32]. Other researchers have also described that hyposplenism, chronic kidney or liver disease and alcoholism were more associated with *S. pneumoniae* meningitis and *H. influenzae* meningitis [21, 27]; acquired immunodeficiency (diabetes mellitus, cancer, the use of immunosuppressive therapy) was more associated with *L. monocytogenes* meningitis; and underlying conditions such as otitis, sinusitis or endocarditis were more associated with *H. influenzae* meningitis and *S. aureus* meningitis [33]. No associations between coexisting conditions, comorbidities and the causative agent were found in our study.

The patients in our study showed classical laboratory signs of bacterial meningitis [14]. Furthermore, we found that the highest CSF white cell count was in the other meningitis group, including *H. influenzae, S. aureus* and *S. pyogenes*; the lowest count was in the *L. monocytogenes* meningitis group, however, without a significant association. The highest CSF protein level was in the other meningitis group, and the lowest CSF glucose level was in the *N. meningitidis* and *S. pneumoniae* groups. A prospective cohort study of 62 patients with *L. monocytogenes* meningitis summarized that CSF abnormalities were not typical for bacterial meningitis in 26% of patients [33]. We found that the highest serum white blood cell count was identified more commonly *in the S. pneumoniae* group, and the lowest platelet count and the highest C-reactive protein level were more commonly seen in patients with *N. meningitidis* meningitis. Our study demonstrated that a lower level of blood glucose was significantly more common in patients with *L. monocytogenes* meningitis. However, a research study in an elderly population with *L. monocytogenes* meningitis revealed high levels of blood glucose [34]. We suggest that the results of our study could be associated with the younger age of patients with *L. monocytogenes* meningitis.

For the treatment of patients with CABM, the most common empiric antibiotic regimens were ceftriaxone and penicillin. During the study period, 96.4% of all *N. meningitidis* (*n* = 55) isolates were sensitive or intermediate to penicillin and 100% were sensitive to ceftriaxone. These results correspond to the national surveillance sensitivity data of invasive *N. meningitidis* issued by the National Public Health Surveillance laboratory: during the 5-year surveillance period from 2014 to 2018 in Lithuania, 93.8% (121/129) of *N. meningitidis* isolates were sensitive or intermediate to penicillin and 100% were sensitive to ceftriaxone [35]. *S. pneumoniae* in our study was sensitive to penicillin in 10 out of 15 patients (67%), and all of them were sensitive to ceftriaxone. The Lithuanian 5-year surveillance period showed that 82.6% (390/472) of invasive *S. pneumoniae* isolates were sensitive to penicillin and 94.5% (273/289) were sensitive to ceftriaxone. In our study, *L. monocytogenes* isolates were all sensitive to penicillin (*n* = 5), and *H. influenzae* isolates were all sensitive to ceftriaxone (*n* = 3).

The median duration of antibiotic therapy was 14 days (IQR 11.0–16). The median time in which antibiotic treatment was started was 40 min (IQR 30.0–90.0) and it did not differ significantly according to the causative agent. CABM is a life-threatening disease, and the timing of antibiotic therapy is crucial. Most scientific reports suggest that delay in antibiotic therapy has been associated with unfavorable outcomes [16, 36, 37]. The antibiotic therapy and duration in our study were as recommended by the European Society of Clinical Microbiology and Infectious Diseases guidelines [14]. Nevertheless, the time before the initiation of antibiotic therapy start was not sufficiently short in all cases. The possible reasons for delayed antibiotic treatment may include the following: 1) the retrospective analysis of medical records could result in an incorrect time of the first antibiotic dose being reported; 2) a transfer from another hospital might have prolonged the time to the first dose of antibiotic; 3) a wait in the diagnostic-treatment sequence (head CT followed by lumbar puncture for CSF study and antibiotics afterwards); and 4) an atypical clinical picture of CABM.

In our population, the majority of patients were treated with dexamethasone, and the mean duration was 6.5 ± 3.5 days. The dose, time of administration of corticosteroids and outcome associated with the implementation of adjunctive corticosteroids, however, differed between studies [4, 38].

Despite advances in diagnosis and treatment, CABM still has a high mortality rate, ranging from 17 to 28%; a requirement of treatment in the ICU; and long-term serious sequalae [1–5, 39]. In our study, hospitalization in the ICU was a significant part of CABM management in many patients. The need for the hospitalization in the ICU was more common in the *N. meningitidis* group corresponding to the insidious course of meningococcal meningitis. Furthermore, the duration of hospitalization in the ICU was significantly longer in the *S. pneumoniae* group. This correlates with the course of pneumococcal meningitis as a risk for severe CNS complications along with hydrocephalus, brain edema, intracranial hemorrhage, cerebral venous and arterial complications and seizures, contributing to mortality and long-term disabilities and the need for a longer duration of hospitalization in the ICU [40].

In our study, death occurred in 5.7% of episodes, and the outcome was unfavorable in 15.7% of episodes and it did not vary significantly according to the etiological agent. The lower case fatality rate than that reported in other studies might have been related to *N. meningitidis* being the main etiological agent [12, 13]. Other studies found that meningitis caused by *S. pneumoniae* had the highest case fatality rates, 20 to 37% for high-income countries and up to 50% for low-income countries, while meningococcal meningitis fatality rates were much lower, between 3 and 10% for high- and low-income countries, respectively [19, 41, 42]. The strongest risk factors in our study for an unfavorable outcome were an older age, coexisting pneumonia and a low platelet count at presentation. According to other studies, impaired consciousness, low white-cell count, a delay in antibiotic therapy initiation and other comorbidities may also lead to a more severe course of meningitis [21, 22, 31, 43].

Our present study has several limitations to note. First, the retrospective nature of the study limited data on detailed medical information for our patients. Second, the number of patients was relatively small, and the study was conducted in just one center. Future prospective studies are necessary to explore diagnostic and therapeutic aspects of patients with CABM and the associations between genetic peculiarities and the increased susceptibility to meningococcal infection in Lithuania.

## Conclusions

The most common causative agent of CABM was *N. meningitidis*, with serotype B being clearly dominant, which corresponds to the high prevalence of invasive meningococcal disease in Lithuania as defined by the latest European Centre for Disease Prevention and Control surveillance data report. Causative agents of CABM did not influence the disease outcome. The strongest risk factors for an unfavorable outcome in patients with bacterial meningitis were older age, coexisting pneumonia and a low platelet count at presentation. Since the introduction of a routine vaccination against meningococcus B for infants in Lithuania in 2018, national vaccination policy may hopefully contribute to a decrease in the incidence of serogroup B meningococcal disease in the Lithuanian population.

## Data Availability

The data are available from the Infectious Diseases Centre, Vilnius University Hospital Santaros Klinikos (VUL SK), but restrictions apply to the availability of these data that were used under protocol for the current study and thus are not publicly available. The datasets generated during the current study are, however, available from the corresponding author on reasonable request and with the permission of VUL SK.

## Abbreviations

4CMenB: Four-component capsular group B meningococcal vaccine
CABM: Community-acquired bacterial meningitis
CSF: Cerebrospinal fluid
ECDC: European Centre for Disease Prevention and Control
EUCAST: European Committee on Antimicrobial Susceptibility Testing
GOS: Glasgow Outcome Scale
ICU: Intensive Care Unit
WBC: White blood cell
